# Evaluation of the Polygenic Risk Score for Alzheimer’s Disease in Russian Patients with Dementia Using a Low-Density Hydrogel Oligonucleotide Microarray

**DOI:** 10.3390/ijms241914765

**Published:** 2023-09-29

**Authors:** Anna Ikonnikova, Anna Morozova, Olga Antonova, Alexandra Ochneva, Elena Fedoseeva, Olga Abramova, Marina Emelyanova, Marina Filippova, Irina Morozova, Yana Zorkina, Timur Syunyakov, Alisa Andryushchenko, Denis Andreuyk, Georgy Kostyuk, Dmitry Gryadunov

**Affiliations:** 1 Center for Precision Genome Editing and Genetic Technologies for Biomedicine, Engelhardt Institute of Molecular Biology, Russian Academy of Sciences, 119991 Moscow, Russia; markelka@rambler.ru (O.A.); elfed0@mail.ru (E.F.); emel_marina@mail.ru (M.E.); mafilippova@mail.ru (M.F.); grad@biochip.ru (D.G.); 2 Mental-Health Clinic No. 1 Named after N.A. Alekseev, Zagorodnoe Highway 2, 115191 Moscow, Russia; hakurate77@gmail.com (A.M.); aleksochneva@yandex.ru (A.O.); abramova1128@gmail.com (O.A.); irinashchelkanova@gmail.com (I.M.); zorkina.ya@serbsky.ru (Y.Z.); sjunja@bk.ru (T.S.); alissia.va@mail.ru (A.A.); denis.s.andreyuk@yandex.ru (D.A.); kgr@yandex.ru (G.K.); 3 Department of Basic and Applied Neurobiology, V. Serbsky Federal Medical Research Centre of Psychiatry and Narcology, Kropotkinsky per. 23, 119034 Moscow, Russia; 4 International Centre for Education and Research in Neuropsychiatry (ICERN), Samara State Medical University, 443016 Samara, Russia; 5 Economy Faculty, M.V. Lomonosov Moscow State University, 119991 Moscow, Russia; 6 Department of Psychiatry, Federal State Budgetary Educational Institution of Higher Education “Moscow State University of Food Production”, Volokolamskoye Highway 11, 125080 Moscow, Russia

**Keywords:** dementia, polygenic risk, Alzheimer’s disease, *APOE*, microarray, genetic risk, neurodegenerative diseases

## Abstract

The polygenic risk score (PRS), together with the *ɛ4* allele of the *APOE* gene (*APOE-ɛ4*), has shown high potential for Alzheimer’s disease (AD) risk prediction. The aim of this study was to validate the model of polygenic risk in Russian patients with dementia. A microarray-based assay was developed to identify 21 markers of polygenic risk and *ɛ* alleles of the *APOE* gene. This case–control study included 348 dementia patients and 519 cognitively normal volunteers. Cerebrospinal fluid (CSF) amyloid-β (Aβ) and tau protein levels were assessed in 57 dementia patients. PRS and *APOE-ɛ4* were significant genetic risk factors for dementia. Adjusted for *APOE-ɛ4*, individuals with PRS corresponding to the fourth quartile had an increased risk of dementia compared to the first quartile (OR 1.85; *p*-value 0.002). The area under the curve (AUC) was 0.559 for the PRS model only, and the inclusion of *APOE-ɛ4* improved the AUC to 0.604. PRS was positively correlated with tTau and pTau181 and inversely correlated with Aβ42/Aβ40 ratio. Carriers of *APOE-ɛ4* had higher levels of tTau and pTau181 and lower levels of Aβ42 and Aβ42/Aβ40. The developed assay can be part of a strategy for assessing individuals for AD risk, with the purpose of assisting primary preventive interventions.

## 1. Introduction

The existence of familial forms of Alzheimer’s disease (AD) suggests that genetic factors play a crucial role in the pathogenesis of this disease. The heritability is 58–79% for late-onset AD and more than 90% for early-onset AD [1].

The most studied but not exhaustive genetic risk factor for AD is the *ɛ4* allele of the *APOE* gene (*APOE-ɛ4*), which encodes apolipoprotein E. This allele increases the risk of AD by 3-fold in heterozygous carriers and 15-fold in homozygous carriers [2]. People who have the *APOE* gene’s *ɛ2* allele (*APOE-ɛ2*) have a lower risk of AD.

The search for reliable genetic risk factors beyond *APOE* has been ongoing for some time. Large international consortia such as the International Genomics of Alzheimer’s Project (IGAP) and the Alzheimer’s Disease Genetics Consortium (ADGC) have been established to examine the genetic basis of Alzheimer’s disease. Genome-wide association studies (GWASs) have accumulated vast amounts of data on AD genetics using samples from tens of thousands of AD patients and healthy donors [3,4]. These studies have identified over 40 loci associated with the disease [5]. This knowledge has significantly contributed to understanding the pathogenesis and polygenic nature of AD, forming a foundation for further research on the molecular processes of the disease’s progression and the development of new therapeutic strategies. However, single-nucleotide polymorphisms (SNPs) typically have a limited impact on disease risk and cannot be utilized as independent prognostic markers. This problem is common among multifactorial diseases. To gauge the impact of genetic factors on disease development, a polygenic risk score (PRS) approach was developed. PRS models estimate the total (multiplicative) impact of several SNPs, which are typically selected based on GWAS using specialized algorithms [6]. Each SNP is assigned a coefficient (often a weighted odds ratio or hazard ratio), and the PRS is computed as the total number of risk alleles multiplied by their corresponding coefficients.

The first PRS model for assessing the risk of developing AD was released in 2005, preceding the large-scale GWAS. This model consisted of nine SNPs, incorporating *APOE-ɛ4* [7]. GWAS data have since given rise to PRS models comprising nineteen to hundreds of thousands of SNPs [8,9,10,11,12,13]. Additional factors such as *APOE* gene alleles, gender, age, and other social and physiological characteristics can also be incorporated.

Studies on PRS models have indicated their correlation with the age and risk of AD and dementia [8,9,10,11,14] as well as their impact on the rate of progression of mild cognitive impairment (MCI) and the risk of its conversion to AD [15,16,17]. Additionally, the relationship between PRS and cognitive function in healthy individuals of various ages has been established [18,19]. Moreover, PRS has been associated with structural and functional brain changes as well as some biochemical indicators related to signs of neurodegeneration [9,20], such as amyloid-β (Aβ) and tau protein deposition [10,21,22,23,24]. Aβ40, Aβ42, phosphorylated tau 181 (pTau181), and total tau (tTau) serve as additional diagnostic markers for AD [25]. Therefore, exploring their correlation with PRS presents an interesting avenue for investigation.

Thus, polygenic models offer a promising approach to identifying individuals at high risk for developing AD. These tests may have practical applications in selecting personalized preventive measures and developing screening strategies. In addition, PRS models could be utilized in the design of clinical trials for AD therapy aimed at preventing disease progression. It is suggested that supplementing study cohorts with individuals who have a high PRS and thus an elevated risk for developing AD may enhance the probability of identifying effective treatment regimens [6,16,26].

It should be noted that most of the research on AD development with PRS has been performed on the European population. Therefore, further studies are required to extend the findings to other populations. In certain instances, polygenic risk models for AD have been investigated in Hispanics, Latin Americans, Asians, and other ancestry groups [9,27,28,29], and promising results have been obtained. In this work, we applied the 21-SNPs PRS model [9] for the first time in patients with dementia and cognitively normal (CN) volunteers from the European part of Russia. Such research is necessary to verify the validity of the model in the population in which it is intended to be used and to specify the risks to individuals as a function of PRS values for further use in clinical practice. The study was carried out using a specially developed DNA microarray-based assay. Furthermore, we explored the correlation of PRS with Aβ and tau proteins in cerebrospinal fluid (CSF).

## 2. Results

### 2.1. Genotyping of Patients with Dementia and CN Volunteers, Association of PRS with Dementia

The study included 348 patients with dementia and 519 cognitively normal volunteers. In total, 867 DNA samples were genotyped for 23 SNPs, including 21 polygenic risk markers according to the model of Tosto et al. [9], as well as rs429358 and rs7412 that define the *ɛ* alleles of the *APOE* gene. A multiplex assay utilizing hybridization on low-density hydrogel microarrays was the primary method for SNP genotyping. The genotype and allele frequency data based on the results of SNP identification are presented in Table S1. The distribution of genotypes for all studied markers conformed to the Hardy–Weinberg equilibrium. Genotyping results for 21 PRS markers from 100 DNA samples (50 from dementia patients and 50 from CN volunteers) were validated using high-resolution melting analysis and selectively confirmed via Sanger sequencing. Additionally, *APOE* alleles were identified via real-time PCR using hydrolysis probes for all samples. The genotyping results obtained by various methods were consistent for 100% of the samples.

For each sample, the PRS was calculated. The PRS values ranged from −1.148 to 1.096. A PRS less than −0.268 corresponded to the first quartile, while a PRS greater than or equal to 0.1725 corresponded to the fourth quartile. The thresholds for the first and top deciles were −0.4598 and 0.3924, respectively.

**Figure 1:**
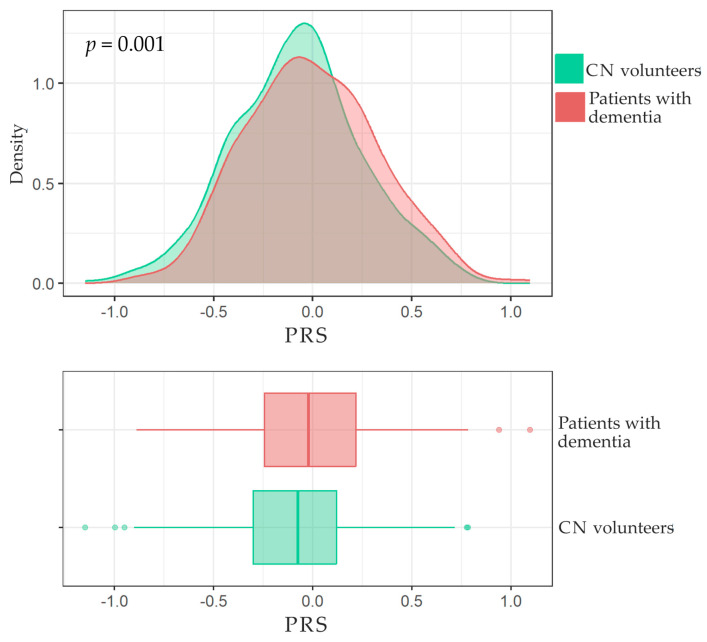
Distribution of PRS in patients with dementia and CN volunteers. The boxplots show the median, with the two hinges corresponding to the first and third quartiles and two whiskers. The upper and lower whiskers extend from the hinges to the largest value no further than 1.5 × IQR from the corresponding hinge (where IQR is the interquartile range). Points beyond the whiskers indicate the outliers.

The distribution of PRS in both patients with dementia and CN volunteers is displayed in Figure 1. A significant difference was observed in the mean PRS between patients with dementia and CN volunteers (*t*-test, *p*-value = 0.001). The logistic regression analysis indicated an association between dementia and both PRS and *APOE-ɛ4* for both homozygotes and heterozygotes, as shown in Table 1. Since the *ɛ2*-allele carriage was not a significant factor in this study, it was not included in the further analysis of how genetic factors impact the risk of dementia.

Adjusted for *APOE-ɛ4*, individuals with a PRS falling within the fourth quartile or tenth decile had a higher risk of dementia compared to those in the first quartile/decile: OR = 1.85 (95% CI = 1.25–2.74; *p*-value = 0.002) and OR = 2.27 (95% CI = 1.22–4.28; *p*-value = 0.01), respectively.

Next, we examined the extent of the impact of *APOE-ɛ4* as a risk factor in individuals with varying PRS values. Compared with non-carriers, carriers of *APOE-ɛ4* with the fourth quartile of PRS had a greater risk of dementia, while the presence of the *APOE-ɛ4* in individuals with the first quartile was not a significant risk factor in our cohort (Table 2).

### 2.2. Predictive Significance of Genetic and Social Factors on the Risk of Dementia

**Figure 2:**
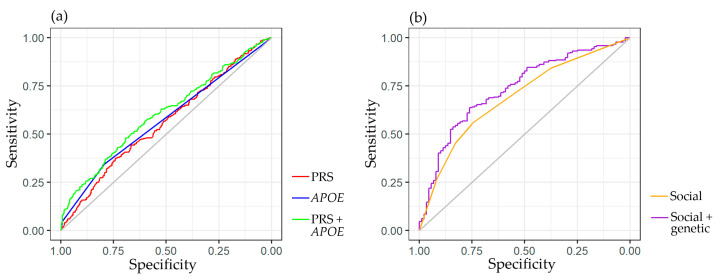
Discriminative ability for predictive models with genetic and social factors. (**a**) ROC curves for predictive models with genetic factors: PRS (AUC = 0.559), *APOE*-*ɛ4* (AUC = 0.573), PRS, and *APOE*-*ɛ4* (AUC = 0.604). (**b**) ROC curves for predictive models with social factors, including family, higher education, and intellectual job (AUC = 0.69), and both social and genetic (PRS and *APOE*-*ɛ4*) factors (AUC = 0.7343).

Receiver operating characteristic (ROC) analysis was conducted on logistic regression models utilizing various predictors. The impact of genetic factors was evaluated across the entire cohort (n = 867), and the ROC curves are displayed in Figure 2a. The area under the curve (AUC) for the PRS model alone was 0.559, while the inclusion of *APOE-ɛ4* improved the AUC to 0.604. The AUC for *APOE-ɛ4* alone was 0.573.

Since detailed questioning of all patients did not occur, a separate analysis was conducted in a group of 347 individuals (88 CN volunteers and 259 patients with dementia) who had available data regarding the presence of a family, higher education, and intellectual work. This analysis considered the impact of social factors in addition to the PRS and *APOE-ɛ4*. When these variables were included, the AUC increased from 0.69 to 0.7343 (Figure 2b). The genetic factors demonstrated a statistically significant improvement in the model (Table S2).

### 2.3. Association of PRS with Amyloid-β and Tau Proteins in Cerebrospinal Fluid

**Figure 3:**
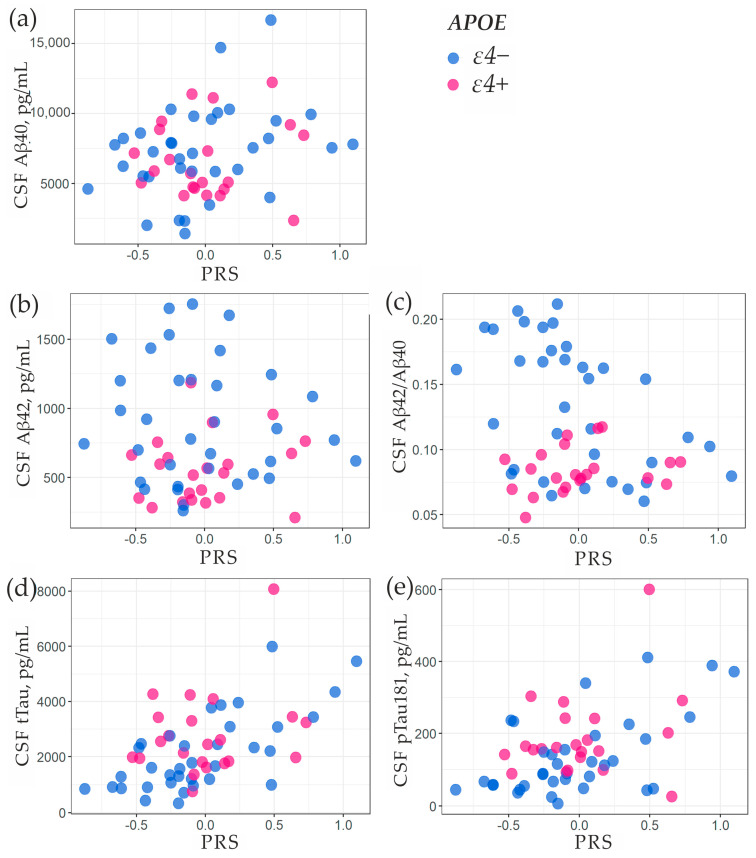
Association of biomarkers in CSF with PRS and the presence of the *ɛ4* allele of the *APOE* gene. (**a**) Aβ40; (**b**) Aβ42; (**c**) Aβ42/Aβ40; (**d**)tTau; (**e**) pTau181.

We examined the impact of genetic factors on the levels of Aβ40, Aβ42, Aβ42/Aβ40, tTau, and pTau181 in CSF obtained from 57 dementia patients. Linear regression was used to determine the association, where PRS and the presence of *APOE-ɛ4* were considered as predictors (Table 3). Dependent variables were logarithmically transformed (ln) for this analysis, allowing the coefficients to represent the percentage change in the concentration of the studied CSF markers (when multiplied by 100). The level of Aβ40 was not affected by the studied genetic factors, while the concentration of Aβ42 was lower in the carriers of *APOE-ɛ4*. The carriers of *APOE-ɛ4* and those with higher PRS had a lower Aβ42/Aβ40 ratio. The levels of both tTau and pTau181 exhibited a positive correlation with the presence of *APOE-ɛ4* and PRS. Figure 3 depicts the relationship between CSF biomarkers and the presence of *APOE-ɛ4* and PRS.

It should be noted that individuals carrying the *ɛ2* allele exhibited a 35.3% higher Aβ42/Aβ40 ratio compared to those without the allele (*p* = 0.006). The presence of this allele did not result in statistically significant differences in other CSF markers.

## 3. Discussion

The polygenic risk scoring approach has become truly innovative in the study of the genetics of multifactorial diseases. Compared to individual SNPs, PRS models often yield highly reproducible results, which holds promise as a prognostic factor. It is advisable to investigate the information content of the developed models in the intended population before implementation. In this study, we employed the PRS model introduced by Tosto et al. [9], which was initially intended for familial late-onset Alzheimer’s disease but subsequently found to share a common architecture with early-onset and sporadic forms [30].

To identify the SNPs included in the utilized PRS model and the *ɛ* alleles of the *APOE* gene, we developed a molecular approach using the established hydrogel microarray technology. The microarray analysis can be completed in less than 12 h. The software that executes the SNP identification algorithm, PRS calculation, and *APOE* allele determination automatically analyzes the microarray hybridization pattern. Compared to next-generation sequencing (NGS) assays, the cost of microarray analysis is significantly lower and comparable to PCR (less than USD 10 per sample). However, microarray-based assays are limited by the incomplete automation of all procedural steps and a lack of standardization that is critical for routine genetic testing in clinical laboratory settings. Nevertheless, several hydrogel microarray-based assays have been approved for in vitro clinical diagnostics by the Russian regulatory agency [31]. In this study, the microarray results were validated via Sanger sequencing and real-time PCR, with SNP identification showing 100% concordance.

The developed assay was applied to investigate the impact of PRS and the *ɛ* alleles of the *APOE* on dementia risk among the residents of the Moscow region in the Russian population, utilizing a sample of 348 individuals diagnosed with dementia and 519 cognitively normal participants. For the first time, the AD polygenic risk model was tested on the European part of the Russian population, revealing its capacity to accurately assess the risk of dementia. The OR of 1.26 (per standard deviation) obtained in this study is equivalent to Tosto et al.’s OR of 1.29 for the European population. Additionally, we achieved similar AUC values (0.559 compared to 0.57) [9].

Of the studied genetic factors, as expected, homozygotes for *APOE*-*ɛ4* had the highest risk of dementia (OR = 7.98). The risk for individuals in the fourth quartile of PRS (OR = 1.85) was approximately equal to the risk for the heterozygous carriers of *APOE-ɛ4* (OR = 1.81). Our data regarding the impact of PRS on disease development risk in *APOE-ɛ4* carriers (Table 2) support previous findings indicating that individuals carrying *ɛ4* with high PRS values exhibit a higher risk [8,32].

Thus, there is compelling evidence that genetic factors, including PRS and *APOE-ɛ4*, influence the risk of developing dementia. However, considering the small AUC values achieved solely using genetic factors, it is improbable that they can be utilized as an independent predictor. In certain studies [8,12], clinical and social factors such as age, gender, *APOE*-*ɛ4*, and education were more crucial for risk assessment than PRS. We showed that AUC values greater than 0.7 were achieved by including social factors in the analysis, such as the presence of higher education, family, and intellectual work. Nevertheless, the statistical significance of PRS as a risk factor for dementia implies that it can enhance the stratification of individuals into risk groups when incorporated into multivariate scales. Such scales should consider biochemical biomarkers that reflect the preclinical stages of the disease [33]. Additionally, it may be valuable to test polygenic risk models that include hundreds of thousands of SNPs (including alleles of the *APOE* gene) and demonstrated an AUC of 0.72–0.84 in the original studies [12,13]. However, these data require further validation in independent cohorts.

According to numerous data, the concentrations of amyloid and tau proteins in the CSF signify neurodegenerative changes linked to AD, and these parameters form the foundation of the ATN classification system (amyloid, tau, and neurodegeneration) for identifying dementia types [34]. Studies have demonstrated decreased levels of Aβ42 and elevated amounts of tau protein in the CSF for patients with AD [29,35]. For amyloid, the Aβ42/Aβ40 ratio is a more informative measure of amyloid plaque burden in the brain than the absolute value of Aβ42 in the CSF [36,37]. We analyzed Aβ40, Aβ42, Aβ42/Aβ40, tTau, and pTau181 for 57 dementia patients. The PRS showed a positive correlation with tTau and pTau181 and an inverse correlation with Aβ42/Aβ40 (Table 3). Therefore, a higher PRS was associated with CSF biomarker levels that are more indicative of AD. Similar associations between polygenic risk and CSF biomarkers have been demonstrated in other studies [22,29,30], although this was not always the case for all biomarkers. In our study, carriers of *APOE-ɛ4* had higher levels of tTau and pTau181 and lower levels of Aβ42 and Aβ42/Aβ40 ratio in the CSF (Table 3), which also reflects an AD-linked pattern. Conversely, the *APOE*-*ɛ2* allele exhibited an association with elevated levels of the Aβ42/Aβ40 ratio.

This study has some limitations. Firstly, the group of studied patients with dementia did not only include patients with AD. Since polygenic risks and *APOE* alleles significantly correlate with AD biomarkers, it can be assumed that the predictive value of genetic factors for AD may be higher. Secondly, this was a case–control study and not a cohort study. Therefore, some social characteristics could depend on the composition of groups of patients and healthy volunteers. Thus, we did not include sex and the presence of children as predictors in the models since they were not representative of the group of CN volunteers. Also, the age of onset of the disease was unknown for most patients; only the age of inclusion was noted.

Future directions lie in prospective cohort studies, which include patients with MCI. The influence of genetic factors needs to be evaluated while considering clinical, social, and biochemical data. These data will help us assess the impact of PRS on MCI patients, with the hydrogel microarray-based technique acting as an auxiliary tool for identifying high-risk individuals.

## 4. Materials and Methods

### 4.1. Study Population 

The study was conducted in adherence to the guidelines of the Declaration of Helsinki. The procedures related to human subject experiments were carried out in accordance with the ethical standards outlined in Protocol No. 1, dated 25 January 2022 and established by the Local Ethics Committee of Mental-health Clinic No. 1 named after N. Alekseev of the Moscow Healthcare Department A. Data from the participants were collected between January 2022 and March 2023.

Patients with dementia (N = 348) were recruited from the gerontological department of Mental-health Clinic No. 1, named after N. Alekseev of the Moscow Healthcare Department A. The inclusion criteria were as follows: patients aged 65 or older, diagnosed with one of the types of dementia (F00, Alzheimer’s disease; F01, vascular dementia; F03, unspecified dementia) according to the International Classification of Diseases (ICD-10). The study included patients with multiple impairments of cortical functions in at least two areas: memory and one of the cognitive functions. These included thinking (planning, programming, abstracting, and establishing cause-and-effect relationships), speaking, practicing, and gnosis. Cognitive impairment causes difficulties in social and work adaptation and in performing everyday tasks. Symptoms manifested against the backdrop of clear consciousness. The duration of the disease was at least 6 months. 

The exclusion criteria included substance abuse and dependence as well as heavy comorbid severe somatic or neurological disorders such as cancer, cirrhosis, chronic lung failure or absence of a lung, tuberculosis, and viral hepatitis. Among the psychoneurological diseases, the exclusion criteria were also as follows: psychogenic pseudodementia, mental retardation, organic brain diseases, dementia due to schizophrenia, brain injury, epilepsy, tumor, HIV and syphilis, and normotensive hydrocephalus. Additionally, renal or liver failure was not permitted.

CN volunteers (N = 519) were recruited from patients who received periodic medical check-ups in outpatient clinic No. 121 (Moscow), and they were matched by age. Participants were excluded if they had psychiatric disorders, a positive family history of psychiatric illness, substance abuse, or severe somatic diseases. All subjects signed a voluntary informed consent form to participate in the experiment.

Some participants (88 CN volunteers and 259 patients with dementia) were evaluated using a structured clinical interview, including MMSE, CDT, and MoCA scales; they answered a detailed questionnaire and participated in neurological examination and neuropsychological testing. Other participants (431 CN volunteers and 89 patients with dementia) did not provide fully detailed descriptive characteristics.

Table 4 displays the characteristics of the study groups. 

### 4.2. DNA Extraction

Genomic DNA was extracted from peripheral blood collected in EDTA-containing tubes using the QIAcube automated DNA extraction system (QIAGEN, Hilden, Germany) or the LumiPure genomic DNA Blood and Buccal Kit (Lumiprobe RUS Ltd., Moscow, Russia) following the manufacturer’s protocols.

### 4.3. Microarray-Based Assay for SNP Genotyping and Calculation of the PRS

In this study, we examined a panel of SNPs that were included in the PRS model proposed by Tosto et al. for AD [9]. The panel included the following SNPs: rs6656401 in the *CR1* gene; rs6733839 in the *BIN1* gene; rs35349669 in the *INPP5D* gene; rs190982 in the *MEF2C* gene; rs9271192 in the *HLA-DRB5/1* gene; rs10948363 in the *CD2AP* gene; rs271 8058 in the *NME8* gene; rs1476679 in the *ZCWPW1* gene; rs11771145 in the *EPHA1* gene; rs28834970 in the *PTK2B* gene; rs9331896 in the *CLU* gene; rs10838725 in the *CELF1* gene; rs7274581 in the *CASS4* gene; rs983392 in the *MS4A6A* gene; rs10792832 in the *PICALM* gene; rs112 18,343 in the *SORL1* gene; rs17125944 in the *FERMT2* gene; rs10498633 in the *RIN-SLC24A* gene; rs8093731 in the *DSG2* gene; rs4147929 in the *ABCA7* gene; and rs3865444 in the *CD33* gene (if the SNP is located in the intergenic region, the closest gene is indicated). Simultaneously, we analyzed rs429358 and rs7412, which determine the *ɛ* alleles of the *APOE* gene.

**Figure 4:**
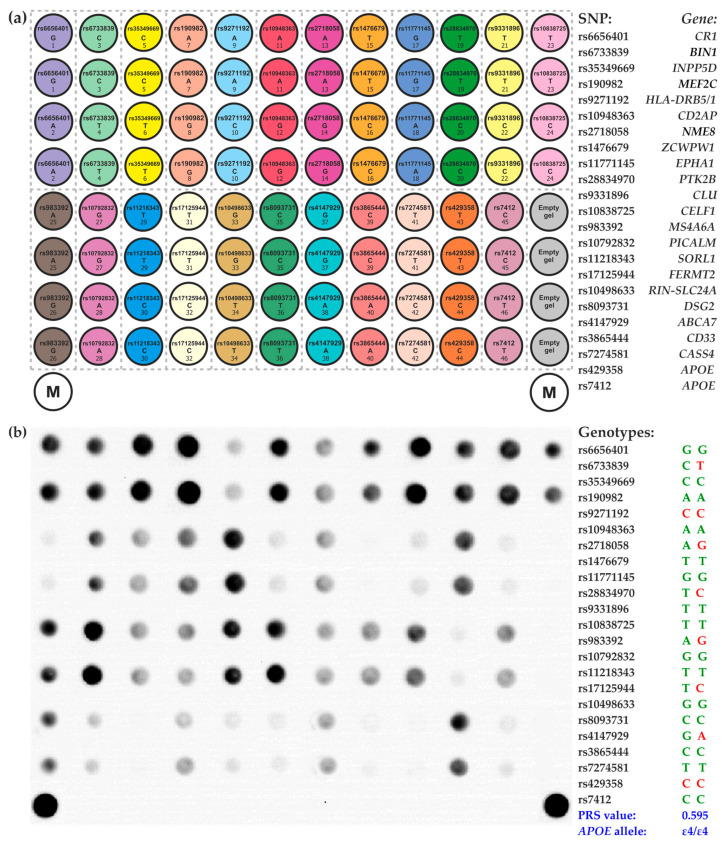
DNA hydrogel microarray for genotyping SNPs associated with Alzheimer’s disease. (**a**) Configuration of the microarray. The analyzed SNPs are indicated within the circles, representing the microarray elements. The microarray included 92 hydrogel elements containing immobilized oligonucleotide probes, two marker elements (M) for image acquisition and processing software, and four empty hydrogel elements for signal normalization. The microarray elements were categorized into 23 groups denoted by distinct colors, which correspond to the analyzed SNPs. Each group comprises four elements, with two of them containing oligonucleotides that match the wild-type allele, while the other two hold probes that correspond to the minor allele. The microarray elements were duplicated to ensure reliable analysis. The right column lists the SNPs and their relevant genes. (**b**) Fluorescence hybridization pattern of the microarray following analysis of genomic DNA from a patient with Alzheimer’s disease. The genotypes obtained for each SNP, PRS, and *APOE* genotype are displayed in the right column.

The identification of 23 SNPs was carried out using a molecular technique that utilizes hydrogel-based oligonucleotide microarray technology developed at the Engelhardt Institute of Molecular Biology, Russian Academy of Sciences [31]. The microarray consisted of a plastic substrate with hydrogel elements (droplets) that were deposited and contained immobilized oligonucleotides. The process of designing and synthesizing oligonucleotides for immobilization, along with the manufacture of microarrays using a copolymerization immobilization method, follows the previously described protocol [38]. Supplementary File S1 provides the sequences of the immobilized probes. Figure 4a displays the layout of the microarray that holds oligonucleotides for identifying 23 SNPs.

The assay protocol involved multiplex PCR to generate mostly single-stranded, fluorescence-labeled DNA fragments, followed by the hybridization of the DNA fragments on a microarray and the acquisition and analysis of fluorescence images. The primer sequences employed for the multiplex PCR and the procedures for carrying out the PCR and microarray hybridization are outlined in Supplementary File S1. The proprietary microarray scanner equipped with original software (Biochip-IMB, Ltd., Moscow, Russia) was used to acquire the fluorescence images and measure signal intensities. The signals of the “wild-type” elements were compared to those of the “minor allele” elements for each group of microarray elements (Figure 4a) to determine their ratio. The genotype was then determined by comparing this ratio to predetermined thresholds, as described before [39]. The *APOE* gene’s *ɛ2/ɛ3/ɛ4* alleles were determined based on whether it had homozygous or heterozygous variants of rs429358 and rs7412.

To calculate the PRS, the model proposed by Tosto et al. [9] was used. The coefficients for the model were obtained from the Polygenic Score (PGS) Catalog (https://www.pgscatalog.org/score/PGS000054/, accessed on 16 August 2023). PRS values were calculated by summing the coefficients multiplied by the number of corresponding effect alleles as follows:

PRS = 0.166 * N_rs6656401_A_ + 0.199 * N_rs6733839_T_ + 0.077 * N_rs35349669_T_ − 0.073 * N_rs190982_G_ + 0.104 * N_rs9271192_C_ + 0.095 * N_rs10948363_G_ − 0.073 * N_rs2718058_G_ – 0.094 * N_rs1476679_C_ − 0.105 * N_rs11771145_A_ + 0.095 * N_rs28834970_C_ − 0.151 * N_rs9331896_C_ + 0.077 * N_rs10838725_C_ − 0.105 * N_rs983392_G_ − 0.139 * N_rs10792832_A_ − 0.261 * N_rs11218343_C_ + 0.131 * N_rs17125944_C_ − 0.094 * N_rs10498633_T_ − 0.315 * N_rs8093731_T_ + 0.14 * N_rs4147929_A_ – 0.062 * N_rs3865444_A_ − 0.128 * N_rs7274581_C_;

where N represents the number of corresponding effect alleles (from 0 to 2).

The fluorescence image examples of the microarray after analyzing DNA samples from AD patients and determining the genotype at each SNP with *APOE* alleles and PRS are shown in Figure 4b.

### 4.4. Verification of the Microarray-Based Genotyping Results

To confirm genotyping results, Sanger sequencing and real-time PCR techniques, including PCR with hydrolysis probes and high-resolution melting analysis, were used. Sanger sequencing was performed using a 3730xl Genetic Analyzer from Applied Biosystems, Foster City, CA, USA. To determine the *ɛ* alleles of the *APOE* gene, we utilized real-time PCR with hydrolysis probes, where probes and primers from Yi et al. were used [40]. High-resolution melting analysis and real-time PCR with hydrolysis probes were carried out using a LightCycler^®^ 96 (Roche, Basel, Switzerland).

### 4.5. Determination of Aβ and Tau Proteins in Cerebrospinal Fluid

CSF was collected from 57 patients via lumbar puncture between 9:00 a.m. and 2:00 p.m. and transferred to sterile polypropylene tubes. The samples were centrifuged at 1500 rpm for 15 min, and the resulting supernatant was divided into 0.5 mL aliquots and stored at −80 °C. The commercially available MILLIPLEX immunoassay (Millipore, Catalog Number HCYTOMAG-60K, Billerica, MA, USA) was utilized according to the manufacturer’s instructions to measure Aβ40, Aβ42, tTau, and pTau181 in the CSF samples. The corresponding commercial kits describe the ranges of detection for the inflammatory parameters, and the analysis was conducted in duplicate. The protein concentration was quantitated in pg/mL.

### 4.6. Statistical Analysis

PRS was analyzed as a quantitative or nominal variable, dividing it into quartiles (1st/2nd–3rd/4th) or deciles (1st/2nd–9th/10th). Logistic regression was utilized to analyze the role of genetic factors in the risk of dementia. ROC analysis was performed to assess the discriminative ability for genetic and social factors. Linear regression was applied to investigate the associations of PRS and *APOE* alleles with CSF biomarkers and MMSE scores. Statistical analysis and visualization were conducted using R version 4.3.1 (R Foundation for Statistical Computing, Vienna, Austria), with the “pROC”, “psych”, and “ggplot2” packages. We checked for correspondence to the Hardy–Weinberg equilibrium using the online service SNPStats (https://www.snpstats.net/, accessed on 21 April 2023) [41] and calculated the adjusted *p*-values using the Benjamini and Hochberg (BH) method. A comparison of logistic regression models, incorporating social and genetic factors, was conducted using JASP software (Version 0.16.3). Statistical significance was determined when the *p*-value was less than 0.05.

Statistical power was assessed using MedCalc^®^ Statistical Software version 22.013 (MedCalc Software Ltd., Ostend, Belgium). We hypothesized that the ROC curve for the studied PRS model will be different from the referenced 0.5, and the expected AUC will be 0.57, according to Tosto et al. [9]. Based on this, as well as on an alpha of 0.05 and a power of 0.8, the sample size was estimated to be at least 249 individuals in each group.

## 5. Conclusions

The selected polygenic risk model for Alzheimer’s disease confirmed its ability to accurately assess the risk of dementia in patients from the European part of the Russian population. Individuals carrying *APOE-ɛ4* with high PRS values exhibit a higher risk of AD development. Associations of the *APOE-ɛ4* and a higher PRS with CSF biomarker levels reflect neurodegenerative changes that are linked to Alzheimer’s disease.

Overall, this study demonstrates the potential of the microarray-based assay for Alzheimer’s disease risk assessment, which may in the future allow for the screening of individuals and the referral of carriers of risk-associated traits to specialized prevention programs.

## Figures and Tables

**Table 1:** Association of PRS and *ɛ*-alleles of *APOE* gene with dementia.

Predictor	OR	95%CI	*p*-Value
PRS (per sd)	1.26	1.09–1.45	0.0014
*APOE*-*ɛ4*_heterozygous	1.81	1.31–2.51	<0.001
*APOE*-*ɛ4*_homozygous	7.98	2.89–28.11	<0.001
*APOE*-*ɛ2*_heterozygous	1	0.67–1.49	0.99
*APOE*-*ɛ2*_homozygous	2.46	0.4–18.99	0.33

PRS, polygenic risk score; OR, odds ratio; CI, confidence interval; sd, standard deviation.

**Table 2:** Association of the *ɛ4*-allele of the *APOE* gene with dementia depending on the PRS quartile.

Predictor	OR	95%CI	*p*-Value
*APOE-ɛ4* + Qu1	1.56	0.89–2.72	0.12
*APOE-ɛ4* + Qu2-3	1.78	0.89–2.64	0.0045
*APOE-ɛ4* + Qu4	3.52	2–6.38	<0.001

OR, odds ratio; CI, confidence interval; Qu, quartile of PRS.

**Table 3:** Linear regression analysis for the associations of biomarkers in CSF with PRS and the presence of the *ɛ4* allele of the *APOE* gene.

	Predictor	Coefficient (β)	SE	*p*-Value	R2 Adjusted
Aβ40, ln	PRS_per sd	0.095	0.065	0.151	0.004
	*APOE_ɛ4+*	−0.051	0.133	0.699	
Aβ42, ln	PRS_per sd	−0.011	0.066	0.867	0.1395
	*APOE_ɛ4+*	−0.447	0.135	0.0016	
Aβ42/Aβ40, ln	PRS_per sd	−0.106	0.044	0.019	0.3025
	*APOE_ɛ4+*	−0.395	0.089	<0.001	
tTau, ln	PRS_per sd	0.323	0.071	<0.001	0.3086
	*APOE_ɛ4+*	0.341	0.145	0.0224	
pTau181, ln	PRS_per sd	0.283	0.098	0.0055	0.1795
	*APOE_ɛ4+*	0.461	0.199	0.0246	

PRS, polygenic risk score; SE, standard error; sd, standard deviation.

**Table 4:** Characteristics of dementia patients and CN volunteers.

	CN Volunteers	Dementia
Sex, female	71.48% (371/519)	65.71% (228/347)
Higher education	57.14% (52/91)	31.06% (91/293)
Family	63.16% (60/95)	46.69% (141/302)
Children	100% (82/82)	55% (165/300)
Intellectual Work	82.6% (76/92)	46.6% (124/266)
Age, mean (sd, min–max)	71.42 (7.19, 59–94)	73.09 (11.4, 35–97)
MMSE, mean (sd, min–max)	28.84 (0.72, 28–30)	9.97 (6.5, 0–24)
CDT mean (sd, min–max)	7.31 (2.82, 1–10)	3.81 (2.93, 0–10)
MoCA mean (sd, min–max)	24.93 (2.71, 18–30)	9.14 (5, 0–22)

## Data Availability

The data presented in this study are available on request from the corresponding author.

## Supplementary Materials

The following supporting information can be downloaded at: https://www.mdpi.com/article/10.3390/ijms241914765/s1.
