# Supplementary material for: Evaluation of the Polygenic Risk Score for Alzheimer’s Disease in Russian Patients with Dementia Using a Low-Density Hydrogel Oligonucleotide Microarray
Source: Int J Mol Sci. 2023 Sep 29;24(19):14765. doi: 10.3390/ijms241914765 (PMC10572681; doi:10.3390/ijms241914765)
Supplement: Supplementary file 1 [file ijms-24-14765-s001.zip › Supplementary File 1.pdf]

Supplementary file 1. Identification of genetic markers associated with the risk of Alzheimer's disease using DNA microarray

Microarray layout:

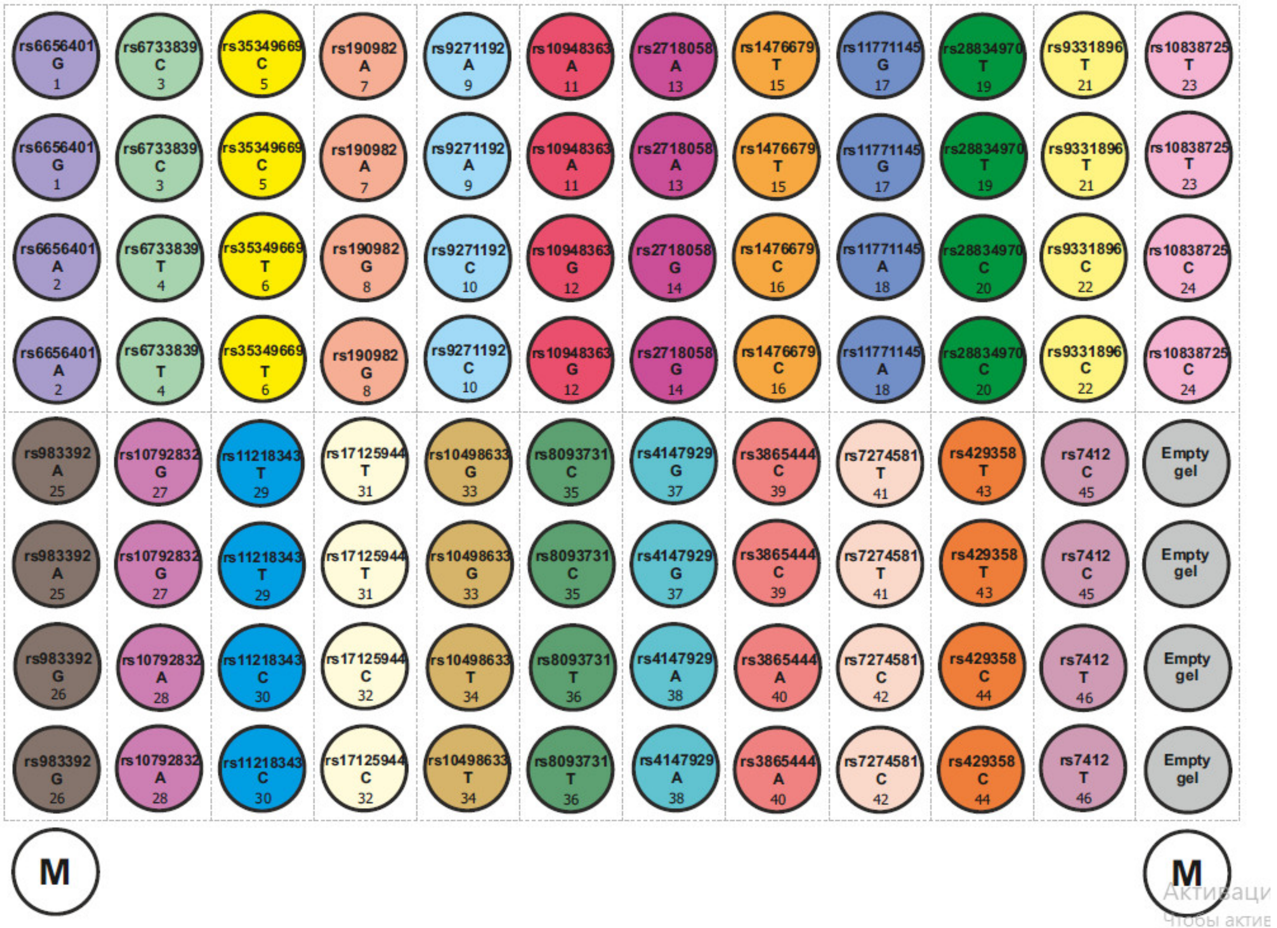

List of oligonucleotides for immobililization on the microarray

	Gene	SNP	Reference Allele	Effect Allele	Sequence, 5'-3'
1	*CR1*	rs6656401	G	A	ATCTTCTCGTCGCCTTC
2					ATCTTCTCATCGCCTTCT
3	*BIN1*	rs6733839	C	T	TTCTTAAAAACACCCTTTTCC
4					TTCTTAAAAATACCCTTTTCC
5	*INPP5D*	rs35349669	C	T	TGATAGCTTCCCACTGAG
6					TGATAGCTTTCCACTGAGT
7	*MEF2C*	rs190982	A	G	CTCAAAGATAAACCAGGACA
8					CTCAAAGATGAACCAGGAC
9	*HLA-DRB5/1*	rs9271192	A	C	CCCCTCTCATAAAAAGTCATA
10					CCCTCTCATACAAAGTCATA
11	*CD2AP*	rs10948363	A	G	GGGCAAAACAAATTTTGTTA
12					GGGCAAAACGAATTTTGTT
13	*NME8*	rs2718058	A	G	TTTTTCCTGTTAATTTCCTATCT

14					TTTTTCCTGTTGATTTCCTATC
15	*ZCWPW1*	rs1476679	T	C	GATCCTGTTATGGACTCCT
16					ATCCTGTTACGGACTCCT
17	*EPHA1*	rs11771145	G	A	TAAACTTTATGAAACGGAATC
18					TAAACTTTATAAAACGGAATCA
19	*PTK2B*	rs28834970	T	C	GTGGAATTGTACAACACTG
20					TGGAATTGCACAACACTG
21	*CLU*	rs9331896	T	C	ACAGCTTTGTGGAGGA
22					ACAGCTTCGTGGAGGA
23	*CELF1*	rs10838725	T	C	GCCAACATTGCACCAC
24					AGCCAACACTGCACCA
25	*MS4A6A*	rs983392	A	G	CTAAAATTCCACATGCAATTTT
26					CTAAAATTCCACGTGCAATTTT
27	*PICALM*	rs10792832	G	A	AAAAATGTAGGAGCAAAACA
28					GAAAAATGTAGAAGCAAAACA
29	*SORL1*	rs11218343	T	C	AGCCGGTAATAGAGTATTTT
30					AGCCGGTAACAGAGTATTT
31	*FERMT2*	rs17125944	T	C	GTTTCAGATCTCCTTGATAG
32					TTTCAGATCCCCTTGATAG
33	*RIN-SLC24A*	rs10498633	G	T	CTCTCAGTCAGTAAACAGTT
34					CTCTCAGTCATTAAACAGTTA
35	*DSG2*	rs8093731	C	T	TCTAATGCCACTCAAGCT
36					TTCTAATGCTACTCAAGCT
37	*ABCA7*	rs4147929	G	A	GCCCATTGTGCCCCT
38					TGCCCATTATGCCCCT
39	*CD33*	rs3865444	C	A	TAAACACCCCATGGATCT
40					TAAACACCCAATGGATCTA
41	*CASS4*	rs7274581	T	C	TCTGGCCTGGCGCG
42					TCTGGCCCGGCGC
43	*APOE*	rs429358	T	C	AGGACGTGTGCGGC
44					GGACGTGCGCGGC
45		rs7412	C	T	TGCAGAAGCGCCTGG
46					GCAGAAGTGCCTGGC

Multiplex PCR

Amplification was carried out in two rounds in the same reaction volume. At the first round, PCR products were generated using locus-specific primers (See Table below) and human genomic DNA as a template. The reaction mixture (25 μl in total) contained 5×PCR Buffer (Asfogen, Ltd, Moscow, Russia), dNTPs (Evrogen, LLC, Moscow, Russia), 5 units of HotTaq Multi DNA polymerase (Asfogen, Ltd, Moscow, Russia), 1 μl of human genomic DNA with typical concentrations of 20-60 ng/μl, and mixture of corresponding forward and reverse primers (see Table below). For the second round of amplification, PCR fragments obtained at

the 1st round were used as a template. A universal Cy5-fluorescently labeled Uni-Cy5 primer was used (see Table below) to yield predominantly single-stranded fluorescently labeled PCR products. PCR was carried out in an S1000 thermal cycler (Bio-Rad, Hercules, CA, USA) under the following conditions: denaturation for 3 min at 95°C, 50 cycles at 95°C - 30 s, 65°C - 30 s, 67°C - 30 s, 69°C - 30 s, 72°C - 20 s, then 50 cycles at 95° C - 20 s, 56° C - 10 s, 72° C - 30 s. The PCR product mixture after the 2nd round of amplification containing predominantly single-stranded fluorescently labeled fragments was used for hybridization on microarray.

List of primers used in multiplex PCR

Gene	SNP	Direction	Sequence, 5′-3′	Concentration in the reaction mixture (mM)
CR1	rs6656401	F	ATCATTTCCTTCTCTGTCTCCATCTTCTC	0,04
		R	tcattggatctcattaGAAGAGCAAAGGACACACACAGAG	0,04
BIN1	rs6733839	F	AAAAGGAAAGAAATCTCTGTTCTGCTTC	0,04
		R	tcattggatctcattaCTAGTCTTCTCTGGTTCCTTCTTCTG	0,04
INPP5D	rs35349669	F	GGTGCAGGCCCACAGTGATA	0,04
		R	tcattggatctcattaCAGCCCACCCCTGATGCTTT	0,04
MEF2C	rs190982	F	CTTTTAAGTGTAGTTTCTATGTGCTCTTCAC	0,04
		R	tcattggatctcattaTTAAAAAGACTTTAATAATTCCTGTCCTGG	0,04
HLA-DRB5/1	rs9271192	F	GGTATCTAAAGTAAAATTCCAATACCCCTCTCA	0,04
		R	tcattggatctcattaGAACCCCAAGGAGCTCTGATAAAGTA	0,04
CD2AP	rs10948363	F	GGATTTGAGTATAAGTTCTAACTTCTTAGGGC	0,04
		R	tcattggatctcattaGGTCATTTTTCTAAACACAACACTTTAGTTCCA	0,04
NME8	rs2718058	F	CTAGGTCTTTTCAGAGAACGAGCATTGG	0,04
		R	tcattggatctcattaAAATTACAACATAAATCAACACAGTTGAAGATAGG	0,04
ZCWPW1	rs1476679	F	CAAAAAACGTCACATGGTACTTAGACTG	0,04
		R	tcattggatctcattaCCATTCTCCCGATCTGTTCTGGC	0,04
EPHA1	rs11771145	F	CCTGACTTAAAACACCACGGAGTG	0,04
		R	tcattggatctcattaGACACCAAAGAATGCATATCAGATGATTCCG	0,04
PTK2B	rs28834970	F	TTATGGATCTGCCTTTTCTGGTCATTCC	0,04
		R	tcattggatctcattaGCTAAGTGAAAGCAGCCGTCGC	0,04
CLU	rs9331896	F	CTCCGGTGGTCCAGACACAG	0,04
		R	tcattggatctcattaCATTCAGCTCTTCCCCTCCCAGG	0,04
CELF1	rs10838725	F	CTTCTGGAGACTGAGGCACGAGAATTA	0,04
		R	tcattggatctcattaCTTGTCGCCCACGATGGAGTA	0,04
MS4A6A	rs983392	F	AGGAACAACAATTTGAACAACAGGTGTTTC	0,04
		R	tcattggatctcattaGTTTTAGACAACTAAGCTTGTGGTAAGTTTCTAC	0,04
PICALM	rs10792832	F	GAGGCCACTTAAAGGAAGTGGGA	0,04
		R	tcattggatctcattaGGCCATCCTTTCTCACACTTACAGC	0,04
SORL1	rs11218343	F	CACTGCACCCAGCCGGTAA	0,04

		R	tcattggatctcattaACTCAAACAAGGATGTGGGAACATGTG	0,04
FERMT2	rs17125944	F	GCTTTTGTTCTGGCCTAGTTTGTGG	0,04
		R	tcattggatctcattaCTGGTACTGGTGCATGATTTTGCC	0,04
RIN-SLC24A	rs10498633	F	CTTCAGGCACTTAGCAGACAAGATGAT	0,04
		R	tcattggatctcattaCCATGGCTGTCCTGTTCCTACCTG	0,04
DSG2	rs8093731	F	GACACTCTAAGGCGGGACTCAGTAATC	0,04
		R	tcattggatctcattaTTCTAATTGGGATGTTAACAGTGGTTTTCAAGC	0,04
ABCA7	rs4147929	F	TGTCCCATTCTCACATTTGCCC	0,04
		R	tcattggatctcattaGCCAGCATTGAGTGTGGAGCA	0,04
CD33	rs3865444	F	CCCAGACACTCACACGGACC	0,04
		R	tcattggatctcattaACACCAGGGCTGATCACTGC	0,04
CASS4	rs7274581	F	GTCAGACCGGGTAAGCAGCTTG	0,04
		R	tcattggatctcattaGCCTCAGCCTCCCAAAGTGGAAG	0,04
APOE	rs429358	F	TGGGCGCGGACATGGAG	0,04
		R	tcattggatctcattaCAGCTCCTCGGTGCTCTGG	0,04
	rs7412	F	GCGATGCCGATGACCTGC	0,04
		R	tcattggatctcattaAGGCGCTCGCGGATGG	0,04
Universal adapter primer Uni-Cy5			taatgagatccaatga	4

F – forward primer$ R – reverse primer

Microarray hybridization

Multiplex PCR product mixture (20 μL) was added to 10 μL of 20×SSPE (Thermo Fisher Scientific, MA, USA) and 10 μl of formamide (Panreac, Spain). Microarray hybridization chambers were filled with 36 μL of resulting mixtures, and microarrays were incubated at 37°C for 6-12 hours. The microarray was washed after removal of the hybridization mixture with 1×SSPE for five minutes twice, then washed with distilled water and air dried.
