# Supplementary material for: Evaluation of the Polygenic Risk Score for Alzheimer’s Disease in Russian Patients with Dementia Using a Low-Density Hydrogel Oligonucleotide Microarray
Source: Int J Mol Sci. 2023 Sep 29;24(19):14765. doi: 10.3390/ijms241914765 (PMC10572681; doi:10.3390/ijms241914765)
Supplement: Supplementary file 1 [file ijms-24-14765-s001.zip › Table S1.pdf]

Table S1. Results of SNP identification using microarray-based assay.

SNP (rsID)	Wild type allele homozygotes, n (%)	Heterozygous, n (%)	Minor allele homozygotes, n (%)	Hardy-Weinberg equilibrium, *p-value*	Benjamini-Hochberg adjusted *p-value*	Minor allele frequency, %
rs6656401	496 (57.2%)	301 (34.7%)	70 (8.1%)	0.015	0.345	8.07
rs6733839	327 (37.7%)	414 (47.8%)	126 (14.5%)	0.83	1	14.53
rs35349669	221 (25.5%)	458 (52.8%)	188 (21.7%)	0.1	1	21.68
rs190982	371 (42.8%)	388 (44.7%)	108 (12.5%)	0.71	1	12.46
rs9271192	393 (45.3%)	381 (44%)	93 (10.7%)	1	1	10.73
rs10948363	513 (59.2%)	313 (36.1%)	41 (4.7%)	0.5	1	4.73
rs2718058	385 (44.4%)	381 (43.9%)	101 (11.7%)	0.65	1	11.65
rs1476679	505 (58.3%)	309 (35.6%)	53 (6.1%)	0.52	1	6.11
rs11771145	318 (36.7%)	398 (45.9%)	151 (17.4%)	0.18	1	17.42
rs28834970	414 (47.8%)	380 (43.8%)	73 (8.4%)	0.3	1	8.42
rs9331896	332 (38.3%)	412 (47.5%)	123 (14.2%)	0.83	1	14.19
rs10838725	426 (49.1%)	355 (40.9%)	86 (41%)	0.34	1	9.92
rs983392	350 (40.4%)	410 (47.3%)	107 (12.3%)	0.46	1	12.34
rs10792832	378 (43.6%)	387 (44.6%)	102 (11.8%)	0.88	1	11.76
rs11218343	793 (91.5%)	74 (8.5%)	0 (0%)	0.4	1	0.00
rs17125944	701 (80.9%)	159 (18.3%)	7 (0.8%)	0.7	1	0.81
rs10498633	541 (62.4%)	280 (32.3%)	46 (5.3%)	0.23	1	5.31
rs8093731	854 (98.5%)	13 (1.5%)	0 (0%)	1	1	0.00
rs4147929	618 (71.3%)	231 (26.6%)	18 (2.1%)	0.6	1	2.08
rs3865444	403 (46.5%)	371 (42.8%)	93 (10.7%)	0.59	1	10.73
rs7274581	726 (83.7%)	136 (15.7%)	5 (0.6%)	0.83	1	0.58
rs429358	640 (73.8%)	206 (23.8%)	21 (2.4%)	0.34	1	2.42
rs7412	738 (85.1%)	124 (14.3%)	5 (0.6%)	1	1	0.58
