# Supplementary material for: Evaluation of the Polygenic Risk Score for Alzheimer’s Disease in Russian Patients with Dementia Using a Low-Density Hydrogel Oligonucleotide Microarray
Source: Int J Mol Sci. 2023 Sep 29;24(19):14765. doi: 10.3390/ijms241914765 (PMC10572681; doi:10.3390/ijms241914765)
Supplement: Supplementary file 1 [file ijms-24-14765-s001.zip › Table S2.pdf]

Table S2. Comparison of logistic regression models including social or social and genetic factors.

Model	Deviance	AIC	BIC	df	X^2	*p-value*	McFadden R^2	Nagelkerke R^2	Tjur R^2	Cox & Snell R^2
H_0	361.594	369.594	384.991	343						
H_1	348.007	360.007	383.103	341	13.587	0.001	0.038	0.059	0.126	0.038

H_0 contains the following predictors: the presence of a family, higher education and intellectual work

H_1, in addition to predictors from H_0, contains genetic factors (polygenic risk score and *APOE-ε4*)
